# “Why didn't they tell us the truth?”

**Published:** 2019-09-10

**Authors:** Elmien Wolvaardt

**Affiliations:** 1 Editor: Community Eye Health Journal, International Centre for Eye Health, London School of Hygiene & Tropical Medicine, UK.

**Admitting our mistakes is part of what defines us as mature and trustworthy human beings. Medical professionals have an even greater responsibility to be truthful to their patients, and it can be devastating – and damaging – if this trust is betrayed.**

Joseph is a soft-spoken accountant. We are on the phone, and he is telling me about his shock at discovering for how long doctors had hidden the truth about his wife's cancer diagnosis.

When his wife, Rose, was diagnosed late last year, the tumour was already large. “I was so shocked. I asked the oncologist: ‘Please tell me this hasn't just come out of nowhere,’” Joseph remembers.

Rose was put on an aggressive treatment regimen, culminating in a session of high-strength radiotherapy just a week before her death.

Still traumatised by the speed of events and the suffering of his beloved wife, the last person Joseph expected to hear from two weeks after Rose's passing was their family doctor. “She told me that the size of the tumour, and the speed of Rose's death, did not make sense. She wanted me to get Rose's notes from the hospital,” says Joseph.

The notes showed that a small tumour was visible in a scan taken more than a year before, when Rose had been admitted with pain. The radiologist on duty, who had been working a 16-hour shift, had missed the tumour.

“It's devastating enough to think that Rose's life could have been saved,” says Joseph. “But every oncologist and specialist who looked at the notes would have seen that scan and known that the cancer had been there for more than a year. Why did no-one say anything to us?”

Perhaps they were afraid of your anger, or that you would sue them, I venture.

“But Rose was not like that; she was strong and kind. She would have understood that mistakes can happen, and she would have accepted this; she would have accepted that the cancer had gone too far.

“I'm not saying it would have been easy for her,” he sighs, “but by not telling us, they took away her choice. If she knew the cancer had gone too far, she would have opted for palliative care, or much less aggressive treatment – enough to be comfortable and make the best of the time she had left.

**Figure:**
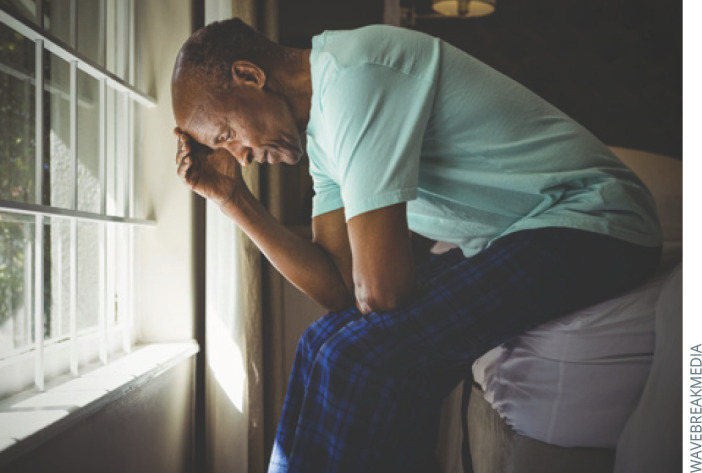
Not being told the truth was devastating for Joseph. (PHOTO POSED BY A MODEL)

“As it was, Rose had nothing of those last five months – and we had nothing of her. Just round after round of chemotherapy, and her being ill for weeks afterwards.

“I kept asking the doctors to speak to me, to explain what was going on, what the purpose was of each new treatment. But nobody would speak to me, not even the nurses who were supposed to be helping us. They kept telling me that they would discuss Rose's case at their next meeting.” He pauses. “Rose never got out of bed again after that last radiotherapy treatment. You should have seen how she looked …,” he says, his voice faltering. “No-one would treat an animal like that.”

There is a long pause. When Joseph speaks again, there is anger in his voice.

“All they cared about was protecting themselves and protecting each other's reputation. What did they talk about at those meetings? About how much longer they could continue hiding the truth from us?”

Joseph is still waiting for answers. Although the hospital's senior leadership team have visited the family at home, they were not able to explain why he and Rose were never told about the mistake, nor why the doctors continued to treat Rose up until the week before her death. The case has now been referred to court.

**“Why didn't they tell us the truth?” – a commentary**

Although this tragic event took place in an oncology clinic, rather than an eye clinic, there are some important lessons for eye care workers:

- The person who made the error was working a 16 hour shift. It is tempting to blame an individual for such a serious event. However, there are wider system failures that led to this situation, and punishing the individual will not prevent a similar event, unless working hours are brought under control.
- Joseph has suffered the loss of his wife, and his suffering has been compounded by the lack of transparency. Reading his story, it is clear that he is much more upset by the hospital's reaction to the mistake than the missed diagnosis itself.
- Because no one gave the patient or her husband a full explanation, they proceeded with aggressive treatment in a vain attempt to cure her and avoid the error coming to light. If we conceal the facts from patients, they cannot be partners in making decisions about their treatment.
- Although keeping quiet about the error may have been intended to avoid litigation, it has led to a lawsuit, which might have been avoided by an early and full disclosure of the missed diagnosis.

– by David Yorston

